# Recurrence Factors and Characteristic Trends of Papillary Thyroid Cancer over Three Decades

**DOI:** 10.1155/2021/9989757

**Published:** 2021-05-10

**Authors:** Waralee Chatchomchuan, Yotsapon Thewjitcharoen, Krittadhee Karndumri, Sriurai Porramatikul, Sirinate Krittiyawong, Ekgaluck Wanothayaroj, Somboon Vongterapak, Siriwan Butadej, Veekij Veerasomboonsin, Auchai Kanchanapitak, Rajata Rajatanavin, Thep Himathongkam

**Affiliations:** Diabetes and Thyroid Center, Theptarin Hospital, Bangkok, Thailand

## Abstract

**Background:**

The prevalence of thyroid cancer is rising worldwide. Although thyroid cancer has a favorable prognosis, up to 20% of patients experienced recurrent disease during the follow-up period. The present study aimed to examine the trend of incidence and factors associated with recurrence and outcomes of papillary thyroid cancer (PTC) in Thai patients over the last 30 years.

**Methods:**

We reviewed the clinical data of all patients with PTC who were treated between 1987 and 2019 at Theptarin Hospital. Clinical characteristics, epidemic trend, factors associated with the persistence/recurrence of the disease, overall disease-specific survival rate, and overall disease-free survival rate were analysed.

**Results:**

A total of 235 patients with PTC who were registered between 1987 and 2019 were reviewed. The mean age was 42.5 ± 14.3 years, with a mean follow-up of 9.5 years. Papillary thyroid microcarcinoma (PTMC) was consistently increased and accounted for 21.4% (50/235) of total cases. The American Thyroid Association (ATA) risk stratification was high in 24% of all PTMCs in the last decade, and 16.0% of these patients experienced local recurrence during the follow-up period. Coexistence with Hashimoto's thyroiditis (HT) was found in one-fifth of the patients with PTC and was correlated with a low recurrence rate (HR: 0.16, *P*=0.013). Only age ≥55 years associated with the persistence/recurrence of the disease. The overall disease-free survival and disease-specific survival rates were 77.4% and 98.3%, respectively.

**Conclusions:**

The prognosis of PTC is generally considered favorable. However, approximately one-fourth of patients with PTMC demonstrated more aggressive clinical behavior, particularly in the last decade of the study. Coexistence of HT contributed to a better prognosis.

## 1. Introduction

Thyroid cancer is the most common form of endocrine cancer, accounting for 3% of all new cancer cases in the United States [1, 2]. Recently, an incidental finding of a small thyroid cancer, known as microcarcinoma, has gained considerable attention [3]. Papillary thyroid cancer (PTC) has generally been documented as an indolent, nonaggressive cancer with a low mortality rate [4]. However, several studies have reported a recurrence in approximately 20% of patients with this disease, in which nearly half of them were identified more than five years after the initial operation [1, 4]. Although recurrence of the disease is not necessarily fatal, it inflicts lifelong and economic burdens to the patients.

Recently, the changing characteristics of papillary thyroid microcarcinoma (PTMC) were observed. PTMC was previously considered as having a good prognostic factor; however, additional studies have revealed otherwise on disease recurrence and metastasis [5]. It is unclear whether this occurrence is because of an increase in thyroid cancer incidence or changes in the disease itself. Recent guidelines have recommended PTMC treatment by lobectomy, unless it has aggressive characteristics [6]. However, a recent meta-analysis showed that less aggressive treatment in patients with PTMC increased the risk of recurrence compared with total thyroidectomy [7]. Identifying clinical characteristics for high-risk patients is essential for effective treatment of PTMC.

Racial disparity also affects cancer incidence and outcomes. Several studies revealed that thyroid cancer incidence was relatively high among Chinese, but lower in South Asian and non-Hispanic White [8–10]. A small cohort showed thyroid cancer tended to be more aggressive, with a higher rate of recurrence and death, in Filipinos compared to other races [11].

The present study aimed to explore the evolution of PTC in Thai patients regarding clinical characteristics, unfavorable risk factors, aggressiveness, and survival over a 30-year period.

## 2. Materials and Methods

### 2.1. Subjects and Data Collection

**Figure 1:**
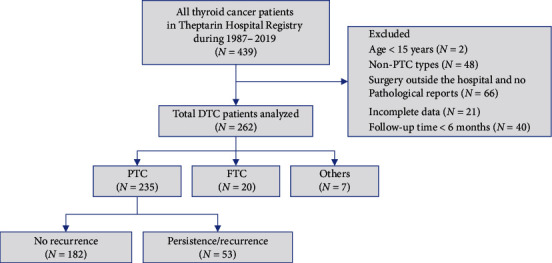
Flowchart depicting the protocol used in this study.

We reviewed the clinical data of all patients diagnosed with PTC who were registered between 1987 and 2019 at Theptarin Hospital, an endocrine center in Thailand. Exclusion criteria included age <15 years, non-PTC types (poorly differentiated thyroid cancer, medullary thyroid cancer, anaplastic thyroid cancer, and other differentiated thyroid cancers), incomplete data, and follow-up time less than six months (Figure 1).

All patients were categorized according to the American Thyroid Association (ATA) risk of recurrence stratification system and tumor-node-metastasis (TNM) staging criteria proposed by the American Joint Committee on Cancer (AJCC), 8^th^ edition [12]. Data on preoperative thyroid ultrasonography, type of operation, and the association of Hashimoto's thyroiditis (HT) were included for analysis. HT was confirmed by pathology. PTC with ≤1 cm diameter was defined as PTMC [6]. Serum thyroid-stimulating hormone (TSH) level, basal and stimulated thyroglobulin levels, neck ultrasonography, dosage and date of radioactive iodine ablation, and whole-body scan were monitored postoperatively. This study was approved by the Ethical Committee of Theptarin Hospital (EC number: 2/2019).

### 2.2. Treatment and Follow-Up

All patients were treated by surgeons performing more than 30 thyroid surgeries per year [13]. Disease monitoring and treatment were determined by the attending endocrinologists. Response to treatment was classified into four categories: excellent, indeterminate, biochemical incomplete, and structural incomplete response, according to the recent guidelines [6]. Time to recurrence was calculated from the day of initial surgery to the day of recurrence confirmed by cytological and/or pathological data. Persistence was defined as incomplete remission after the first surgery within one year.

### 2.3. Statistical Analysis

Statistical Package for the Social Sciences (version 21.0; IBM, New York, USA) was used for statistical analysis. Data were presented as means ± standard deviations or medians ± interquartile ranges (IQRs). Chi-square test was used to compare different categories. Differences in the mean between groups were analysed using a *t*-test or ANOVA test. Cox proportional hazards model was used to perform univariate and multivariate analyses to analyze the factors associated with recurrence. Potential risk predictors were age, sex, body mass index (BMI), ATA risk, coexistence of HT, tumor size, extrathyroidal extension, and multifocality. The disease-free survival rate was estimated using the Kaplan–Meier method and compared by using the log-rank test. All *P* values were two-sided. *P* < 0.05 was considered statistically significant.

## 3. Results

### 3.1. Clinical Characteristics and Trends of Thyroid Cancer

Table 1 summarizes the 235 cases that were included in the study. Approximately one-fifth of all cases of PTC were PTMC (*n* = 50, 21.4%). Coexisting thyroid disease, including Graves' disease and HT, was found in 23 (9.8%) and 46 (19.6%) of the total patients, respectively. Patients with persistent/recurrent disease were older than those with remission, with the mean age of 44.7 ± 16.3 years vs. 41.8 ± 13.6 years, respectively. The median follow-up was 9.5 years (range: 0.5–31.3 years).

In total, 201 (85.5%) patients presented with a neck mass. Physical examination revealed thyroid tumors in 30 asymptomatic patients (12.8%). Two patients (0.9%) had abnormal neck ultrasonography. The remaining patients (*n* = 2, 0.9%) had other initial presentations, including axillary lymph node enlargement and an incidental finding from a thyroidectomy specimen.

Of 235 patients, 81.7% underwent surgery at our hospital, while the rest were referred from other hospitals for further management after the initial operation. Total or near-total thyroidectomy was the most performed procedure (*n* = 226, 96.2%). Most patients (*n* = 231, 98.3%) received at least one dose of postoperative radioactive iodine. Transient and permanent hypoparathyroidism were found in 35.3% (*n* = 83) and 4.7% (*n* = 11) of patients, respectively. Only 2.1% of patients experienced permanent recurrent laryngeal nerve injury. Most patients (*n* = 205, 87.2%) received thyroid hormone suppression therapy, with serum TSH levels < 0.01 mU/L.

Table 2 shows the prevalence rate of thyroid cancer divided by decade. The incidence of PTMC increased over the study time, from 15.2% in 1987–1996 to 24.8% in 2007–2019, but without statistical significance, while the age, sex, BMI, and TNM stage revealed no differences. A significant increase of the high-risk ATA group among overall PTC patients was observed (13.0% in 1987–1996 vs. 34.4% in 2007–2019).

**Figure 2:**
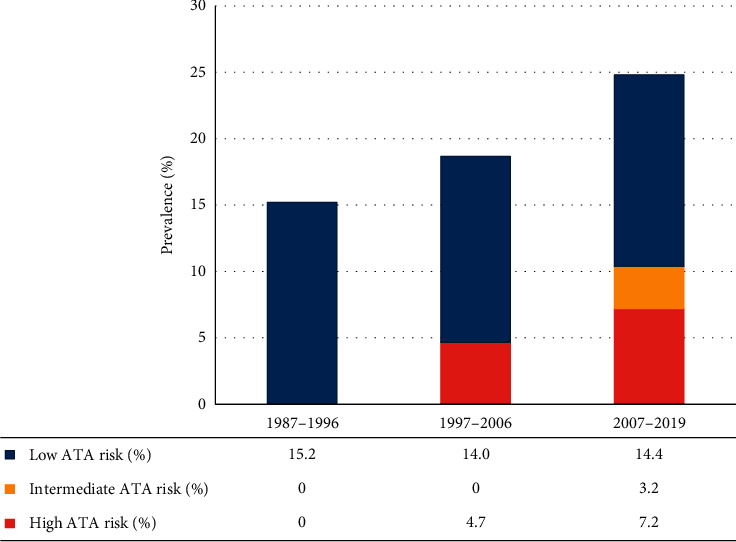
The prevalence of papillary thyroid microcarcinoma classified by ATA risk in each decade of the study period.

Additionally, a subgroup analysis of PTMC showed that 24% of all PTMCs belonged to the high-risk ATA group. This trend continued to rise over the study period. In the first decade of the study, all patients with PTMC were classified as low-risk ATA group, whereas in the second and third decades of the study, 4.7% and 7.2% of patients with PTMC were classified as high-risk ATA group, respectively (Figure 2). Furthermore, 16.0% of all PTMC patients developed a local recurrence.

### 3.2. Follow-Up and Clinical Response Status after Initial Treatment

Most patients had an excellent response (*n* = 151, 64.3%). The biochemical incomplete and structural incomplete response rates were 17.0% (*n* = 40) and 8.5% (*n* = 20), respectively. After completing initial treatment, 53 patients (22.6%) developed persistent/recurrent cancer during the follow-up period. Overall disease-free survival was 77.4%. A low mortality rate was observed in 11 patients (4.7%) who died during the study period. Only four patients (1.7%) died from a cancer-specific cause.

### 3.3. Factors Associated with Persistent and Recurrent Disease

**Figure 3:**
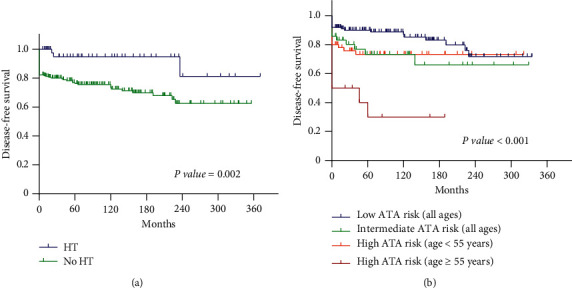
Disease-free survival curves of patients with papillary thyroid cancer based on the following classifications: (a) coexistence of Hashimoto's thyroiditis (HT); (b) ATA risk category.

Table 3 summarizes the potential risk factors of various clinicopathological characteristics on persistence and recurrence of cancer. Univariate analysis showed that age ≥55 years, high ATA risk, and tumor size >4 cm were associated with an increased risk of recurrence/persistence of cancer, whereas gender, BMI >27 kg/m^2^, multifocality, and extrathyroidal extension had no effects. In multivariate analysis, only age ≥55 years was a significant predictor of a poor outcome. Further analysis revealed that higher recurrent rate was found only in patients at age ≥55 years in the high-risk ATA group when compared to patients <55 years old (*P*=0.001). This effect was not found in patients with low and intermediate ATA risk groups (*P*=0.270 and 0.051, respectively). Coexistence of HT was revealed to be a protective factor in both univariate and multivariate analyses.  Supplementary 1 shows the characteristics of PTC according to the coexistence of HT. Disease-free survival curves of the HT and ATA risk category are shown in Figure 3.

## 4. Discussion

Our main finding consistently showed that the prevalence rate of thyroid cancer has risen, particularly in the past decade due to an increase in the incidence of PTMC. A recent study reported that PTMC contributed to 30% of all cases of PTC [1], supported our finding (24.8%). An increase in incidental findings from imaging and the need for a diagnosis have contributed to this observation. Moreover, genetic mutation and carcinogenesis from an increase in radiation exposure, dietary changes, and the use of chemical fertilizers or genetically modified food may also be responsible [14]. However, recurrence and mortality rates have been growing despite early diagnosis and treatment. A recent large cohort study showed that the mortality rate of thyroid cancer has sharply increased during the past decade [1]. Possible explanations may include an underestimation of the aggressiveness of the cancer from under-risk stratification or mutation of the tumor.

According to current guidelines, PTMC has a good prognosis. However, a previous study conducted during the early 2000s reported that 14% of PTMCs were aggressive [15]. More recent study in 2019 indicated that up to 19% of PTMCs had advanced features, including lymph node metastasis, extrathyroidal extension, lymphovascular invasion, and distant metastasis [5]. These trends and clinical findings were similar to those found in our study. The 8^th^ edition AJCC guidelines do not suggest fine needle aspiration for PTMCs unless they have clear evidence of aggressive behavior and highly suspicious ultrasonography findings. This recommendation was based on the data from 1940 to 2000, which stated that PTMC had an excellent prognosis and a very low rate of recurrence (2–6%) [16]. However, regarding the upward trend of PTMC aggressiveness, an individual tailored approach to treatment is essential.

Older age and recurrent rate have found a linear correlation. Chereau et al. indicated worse prognosis with increasing age, especially in patients >75 years old. The recurrence rate increased almost twofold in patients >75 years old compared to patients <65 years old (6.2% vs. 11.7%, respectively) [17]. In addition, a recent study by Kauffmann et al. showed that older patients had a higher five-year mortality rate (hazard ratio = 2.3) compared to patients <45 years old. This effect was independent of gender, race, number of comorbidities, type of operation, hospital volume, or insurance coverage [18]. However, previous study showed that the ATA risk category applying with age showed differences in survival [19]. Similar to our results, only high-risk ATA category with age at cutoff 55 years showed significantly higher recurrent rate, while this effect was not found in low and intermediate ATA risk category. Therefore, very old patients should be considered high-risk patients, and age should be applied with the ATA risk category to improve the stratification system.

Our study showed that patients with PTC and coexisting HT had favorable outcomes. HT has long been debated whether it is a risk factor for thyroid cancer and contribution of the prognosis [20]. It is believed that chronic inflammation of the thyroid gland and high TSH levels, typically found in patients with HT, might be associated with neoplastic changes [21, 22]. Several observational studies and meta-analyses have shown that HT was associated with PTC incidence [23–25] and better prognosis regarding less lymph node involvement, less extrathyroidal extension, smaller tumor size, and longer survival [25, 26]. In a large retrospective study with a nine-year follow-up, the cancer-specific mortality and recurrence rates were lower in patients with coexisting PTC and HT compared to those without HT (2.2% vs. 4.6% and 4.3% vs. 14%, respectively) [27]. Tumor cells can trigger both innate and noninnate immunity, which may lead to an antineoplastic immune response [28].

Recurrence/persistence of PTC has been reported to range from 8.4% to 32% [29], which was similar to our results. These variations might result from different initial approach methods, severity of the disease, treatment, race, and follow-up duration. In this study, most of our patients had a serum TSH level <0.01 over the treatment period which might affect the treatment outcomes.

### 4.1. Limitation of the Study

Our study has some limitations because of its retrospective nature and possible selection bias. Additionally, the data might not be fully representative of the total population because of the limited sample size. However, this study provides the trends and clinical characteristics of PTC over a 30-year period.

## 5. Conclusions

The incidence and aggressiveness of thyroid cancer have increased, and increased incidence of PTMC has contributed to this trend. The outcome of PTMC may not be favorable. Treatment should be in accordance with risk stratification. Coexistence with HT is considered a good prognostic, and age ≥55 years is associated with poorer outcomes.

## Figures and Tables

**Table 1:** Demographic data of 235 papillary thyroid cancer patients.

	Total (*n* = 235)	No recurrence (*n* = 182)	Persistence/recurrence (*n* = 53)	*P* value
Age at initial diagnosis (years)	42.5 ± 14.3	41.8 ± 13.6	44.7 ± 16.3	0.180
<55	189 (80.4)	154 (84.6)	35 (66.0)	
55–70	38 (16.2)	23 (12.6)	15 (28.3)	
>70	8 (3.4)	5 (2.8)	3 (5.7)	
Female (%)	192 (81.7)	153 (84.1)	39 (73.6)	0.082
BMI (kg/m^2^)	22.1 ± 3.8	22.9 ± 3.8	22.9 ± 3.6	0.925
ATA risk (%)				0.013
Low	124 (52.8)	105 (57.7)	19 (35.8)	
Intermediate	42 (17.8)	31 (17.0)	11 (20.8)	
High	69 (29.4)	46 (25.3)	23 (43.4)	
Size (cm)	2.3 ± 1.4	2.2 ± 1.3	2.7 ± 1.7	0.031
≤1	50 (21.4)	42 (23.1)	8 (15.4)	
>1–2	76 (32.0)	57 (31.3)	19 (34.6)	
>2–4	90 (38.5)	72 (39.6)	18 (34.6)	
>4	19 (8.1)	11 (6.0)	8 (15.4)	
Extrathyroidal extension (%)	18 (7.7)	12 (6.6)	6 (11.5)	0.238
8^th^ AJCC staging (%)				<0.001
I	211 (89.7)	171 (94.0)	40 (75.4)	
II	19 (8.1)	10 (5.5)	9 (17.0)	
III	2 (0.9)	0	2 (3.8)	
IV	3 (1.3)	1 (0.5)	2 (3.8)	
Follow-up time (years)	9.5 ± 7.7	9.4 ± 7.6	9.7 ± 7.8	0.769
Coexistence of Hashimoto's thyroiditis (%)	46 (19.6)	44 (24.2)	2 (3.8)	<0.001

AJCC: the American Joint Committee on Cancer.

**Table 2:** Demographic data of papillary thyroid cancer in each decade of the study period.

	1987–1996 (*n* = 46)	1997–2006 (*n* = 64)	2007–2019 (*n* = 125)	*P* value
Age at initial diagnosis (years)	38.6 ± 13.2	43.6 ± 15.2	43.3 ± 14.0	0.128
Female (%)	41 (89.1)	48 (75.0)	103 (82.4)	0.160
BMI (kg/m^2^)	22.3 ± 3.2	23.1 ± 3.9	23.0 ± 3.9	0.502
ATA risk (%)				0.036
Low	29 (63.1)	37 (57.8)	58 (46.4)	
Intermediate	11 (23.9)	7 (10.9)	24 (19.2)	
High	6 (13.0)	20 (31.3)	43 (34.4)	
8^th^ AJCC staging (%)				0.597
I	43 (93.5)	57 (89.1)	111 (88.8)	
II	3 (6.5)	5 (7.8)	11 (8.8)	
III	0	0	2 (1.6)	
IV	0	2 (3.1)	1 (0.8)	
Coexistence of Hashimoto's thyroiditis (%)	7 (15.2)	10 (15.6)	29 (23.2)	0.328
PTMC (%)	7 (15.2)	12 (19.0)	31 (24.8)	0.348

**Table 3:** Potential factors of persistent/recurrent papillary thyroid cancer.

	Univariate analysis			Multivariate analysis		
Factor	HR	95% CI	*P* value	HR	95% CI	*P* value
Age ≥55 years	2.83	1.41–5.68	0.003	2.67	1.27–5.61	0.010
Male	1.89	0.91–3.92	0.086			
High ATA risk	2.27	1.20–4.29	0.012	1.73	0.86–3.45	0.122
Tumor size >4 cm	3.24	1.45–7.22	0.004	2.16	0.91–5.12	0.081
Coexistence of Hashimoto's thyroiditis	0.12	0.03–0.53	0.005	0.16	0.04–0.68	0.013
BMI >27 kg/m^2^	0.84	0.34–2.04	0.696			
Multifocality	1.52	0.78–2.98	0.219			
Extrathyroidal extension	1.84	0.66–5.19	0.244			

## Data Availability

The data that support the findings of this study are restricted to the Institutional Review Board of Theptarin Hospital. Due to the privacy of patients, the data are available from the corresponding author upon reasonable request.

## Supplementary Materials

Supplementary Materials Supplementary 1: demographic data of 235 papillary thyroid cancer patients according to the coexistence of HT.
