# Supplementary material for: Recurrence Factors and Characteristic Trends of Papillary Thyroid Cancer over Three Decades
Source: Int J Endocrinol. 2021 May 10;2021:9989757. doi: 10.1155/2021/9989757 (PMC8131146; doi:10.1155/2021/9989757)
Supplement: Supplementary Materials — Supplementary 1: demographic data of 235 papillary thyroid cancer patients according to the coexistence of HT. [file 9989757.f1.pdf]

Supplementary 1. Demographic data of 235 papillary thyroid cancer patients

	Total (n= 235)	No HT (n= 189)	HT (n= 46)	*P-value*
**Age at initial diagnosis (years)**	42.5±14.3	43.3±14.7	39.0±12.2	0.07
**<55**	189(80.4)	150(79.4)	39(84.8)	
**55-70**	38(16.2)	31(16.4)	7(15.2)	
**>70**	8(3.4)	8(4.2)	0	
**Female (%)**	192(81.7)	150(79.4)	42(91.3)	0.060
**BMI (kg/m^2)**	22.1±3.8	23.0±3.9	22.5±3.2	0.377
**ATA risk (%)**				0.056
**Low**	124(52.8)	96(50.8)	28(60.9)	
**Intermediate**	42(17.8)	31(16.4)	11(23.9)	
**High**	69(29.4)	62(32.8)	7(15.2)	
**Size (cm.)**	2.3±1.4	2.3±1.5	2.2±1.0	0.624
**≤1**	50(21.4)	44(23.4)	6(13.0)	
**>1-2**	76(32.0)	57(29.8)	19(41.3)	
**>2-4**	90(38.5)	70(37.2)	20(43.5)	
**>4**	19(8.1)	18(9.6)	1(2.2)	
**Extrathyroidal extension (%)**	18(7.7)	18(9.6)	0	0.029
**8th AJCC staging (%)**				0.831
**I**	211(89.7)	170(89.9)	41(89.1)	
**II**	19(8.1)	15(7.9)	4(8.7)	
**III**	2(0.9)	2(1.1)	0	
**IV**	3(1.3)	2(1.1)	1(2.2)	
